# Human placenta and trophoblast development: key molecular mechanisms and model systems

**DOI:** 10.1007/s00018-019-03104-6

**Published:** 2019-05-03

**Authors:** Martin Knöfler, Sandra Haider, Leila Saleh, Jürgen Pollheimer, Teena K. J. B. Gamage, Joanna James

**Affiliations:** 1 Reproductive Biology Unit, Department of Obstetrics and Gynaecology, Medical University of Vienna, Währinger Gürtel 18-20, 5Q, 1090 Vienna, Austria; 2 Department of Obstetrics and Gynaecology, Faculty of Medical and Health Sciences, University of Auckland, Auckland, New Zealand

**Keywords:** Placenta development, Chorionic villus, Trophoblast stem cell, Trophoblast differentiation, Mesenchymal cell

## Abstract

Abnormal placentation is considered as an underlying cause of various pregnancy complications such as miscarriage, preeclampsia and intrauterine growth restriction, the latter increasing the risk for the development of severe disorders in later life such as cardiovascular disease and type 2 diabetes. Despite their importance, the molecular mechanisms governing human placental formation and trophoblast cell lineage specification and differentiation have been poorly unravelled, mostly due to the lack of appropriate cellular model systems. However, over the past few years major progress has been made by establishing self-renewing human trophoblast stem cells and 3-dimensional organoids from human blastocysts and early placental tissues opening the path for detailed molecular investigations. Herein, we summarize the present knowledge about human placental development, its stem cells, progenitors and differentiated cell types in the trophoblast epithelium and the villous core. Anatomy of the early placenta, current model systems, and critical key regulatory factors and signalling cascades governing placentation will be elucidated. In this context, we will discuss the role of the developmental pathways Wingless and Notch, controlling trophoblast stemness/differentiation and formation of invasive trophoblast progenitors, respectively.

## Introduction

Formation of the placenta, the unique exchange organ between mother and fetus, is essential for successful human pregnancy and fetal health. Derived from extraembryonic tissues, the placenta rapidly develops during the first weeks of gestation dynamically changing its structure and function [1, 2]. Throughout pregnancy the placenta fulfils a plethora of tasks ranging from physiological adaption of the mother to immunological acceptance, nourishment and support of the developing embryo. Placental villi, bathed in maternal blood, represent the transport units of the organ, delivering nutrients and oxygen to the developing fetus and clearing its waste products. During the 9 months of gestation these villi undergo dynamic morphological changes. Mesenchymal villi of early pregnancy develop into highly vascularized structures, efficiently extracting substances from the maternal circulation [3]. By term, the extensive branching morphogenesis of villi creates an overall epithelial surface of about 12–14 m^2^ ensuring adequate nutritional supply, at a time when the fetus shows high growth rates. Besides fulfilling the needs of the developing fetus, the placenta also profoundly changes the metabolism of the mother by secreting numerous hormones into the maternal blood stream [4]. These hormones affect most maternal tissues and organs, and effectively modulate the maternal physiology to promote the maintenance of pregnancy, mobilization of nutrients, parturition and lactation. Moreover, some placental hormones are also released into the fetal circulation thereby regulating fetal development, growth and timing of delivery [5, 6].

Failures in placental formation, can compromise embryonic growth and development. Indeed, abnormal placentation is a feature of diverse pregnancy complications such as miscarriage, stillbirth, pre-term labour, intrauterine growth restriction (IUGR) and preeclampsia [7–13]. Albeit these disorders can have multiple causes, including fetal aberrations and maternal factors [14, 15], placental defects, inappropriate adaption and remodelling of the uterine vascular bed, and as a possible consequence malperfusion occurs in a considerable number of cases, particularly in severe IUGR and early onset preeclampsia [16–19]. Accordingly, transcriptomic analyses of preeclamptic placentae revealed different subclasses of the disease with specific gene modules for placental dysfunction [20–22]. Disorders with underlying placental abnormalities not only increase morbidity and mortality of mother and fetus, but may also negatively affect long-term health [23, 24]. Mothers with preeclampsia and/or infants born with growth restriction have a higher risk for developing types 2 diabetes, hypertension or cardiovascular disease in later life [25–27]. In addition, developmental programming of the embryo, due to structural, developmental or functional defects of the placenta, may also predispose the fetus to a variety of other chronic adult diseases [28]. For example, abnormal fetal growth impairs neuronal development elevating the risk for psychiatric disorders in adulthood [29].

Considering the complex role of the placenta in fetal–maternal communication, it may not be surprising that its diverse functions at different stages of normal and abnormal pregnancies remain poorly understood. Within the first weeks of pregnancy the human placenta generates epithelial trophoblast with diverse biological roles including attachment of the conceptus to the uterine wall, establishment of early histiotrophic nutrition (nourishment of the fetus by decidual glandular secretions) and adaption of the maternal uterine vasculature [30, 31]. Different types of trophoblasts, including stem cells, progenitors and differentiated subtypes with multiple functions develop [32]. However, their specific roles, particularly in the context of pregnancy disorders, remain largely elusive. Our ability to understand how inadequate placentation contributes to pregnancy disorders is confounded by the fact that the pathogenesis of these disorders develops during the first trimester of pregnancy, when availability of placental tissue for in vitro studies is ethically more limited, and crucially, when we cannot accurately predict which placentae would have gone on to develop pathology later in gestation. Furthermore, culture conditions that allow for self-renewal and long-term propagation of primary trophoblasts, a prerequisite for detailed molecular investigations, have only been recently established [33, 34].

As a result, our knowledge of the functional aspects of placental development is largely based on investigations performed in mice. In diverse knock-out studies key regulatory genes have been unravelled, some of which are also expressed in the human placenta [35–38]. Although specific trophoblast lineages considerably differ between mice and men, both species show haemochorial placentation, resulting in direct contact of maternal blood with fetal-derived trophoblasts. Mouse studies have not only identified specific regulators of major biological processes in the placenta, such as placental vascularization, labyrinth and junctional zone formation and function, but also delineated the importance of the decidualized maternal endometrium (decidua) in governing placental development, trophoblast differentiation and fetal growth [39]. Notably, correct specification and functionality of distinct placental trophoblast subtypes at early stages of development could be crucial for subsequent organ development in the fetus itself. Studies in mice, carrying mutations that provoke lethality around mid-gestation suggest that the preceding placental defects could be causative for fetal demise [40]. In particular, failures in heart, neuronal and vascular development were found to be associated with placental abnormalities. Notably, restoring gene function in the placenta compensated for the embryonic defects in these mutants. An equivalent role of the human placenta in subsequent organogenesis seems likely. For example, failures in adapted perfusion of the placenta could alter haemodynamics in the feto-placental circulation and thereby impair cardiac development [41].

However, despite some similarities, considerable differences haven been noticed between murine and human placental development and structure. Besides deviations in gross morphology and specific trophoblast cell types, blastocysts implant differently in mice, trophoblast invasion is very shallow and remodelling of uterine arterial vessels largely depends on maternal factors [42]. Moreover, key regulators of placental development differ between mouse and men [37], as also outlined below, making the mouse an imperfect model of human placentation. Hence, establishment and further improvement of appropriate human model systems are highly warranted. Considering the crucial role of the placenta in pregnancy complications and long-term health, better insights into molecular mechanisms of human implantation and early placental development should advance options for therapeutic treatment of pregnancy pathologies. However, the critical steps of normal and pathological placentation have hardly been elucidated. Herein, we summarize our current knowledge of human placental development and its underlying mechanisms. Structural changes of the human placenta throughout pregnancy and the specific roles of trophoblast subtypes will be discussed. Further, we will focus on different stem and progenitor cells present in chorionic villi and elucidate key regulatory pathways controlling placentation, trophoblast development and differentiation.

## Development and functional properties of the early chorionic villus and its different cell types

**Fig. 1:**
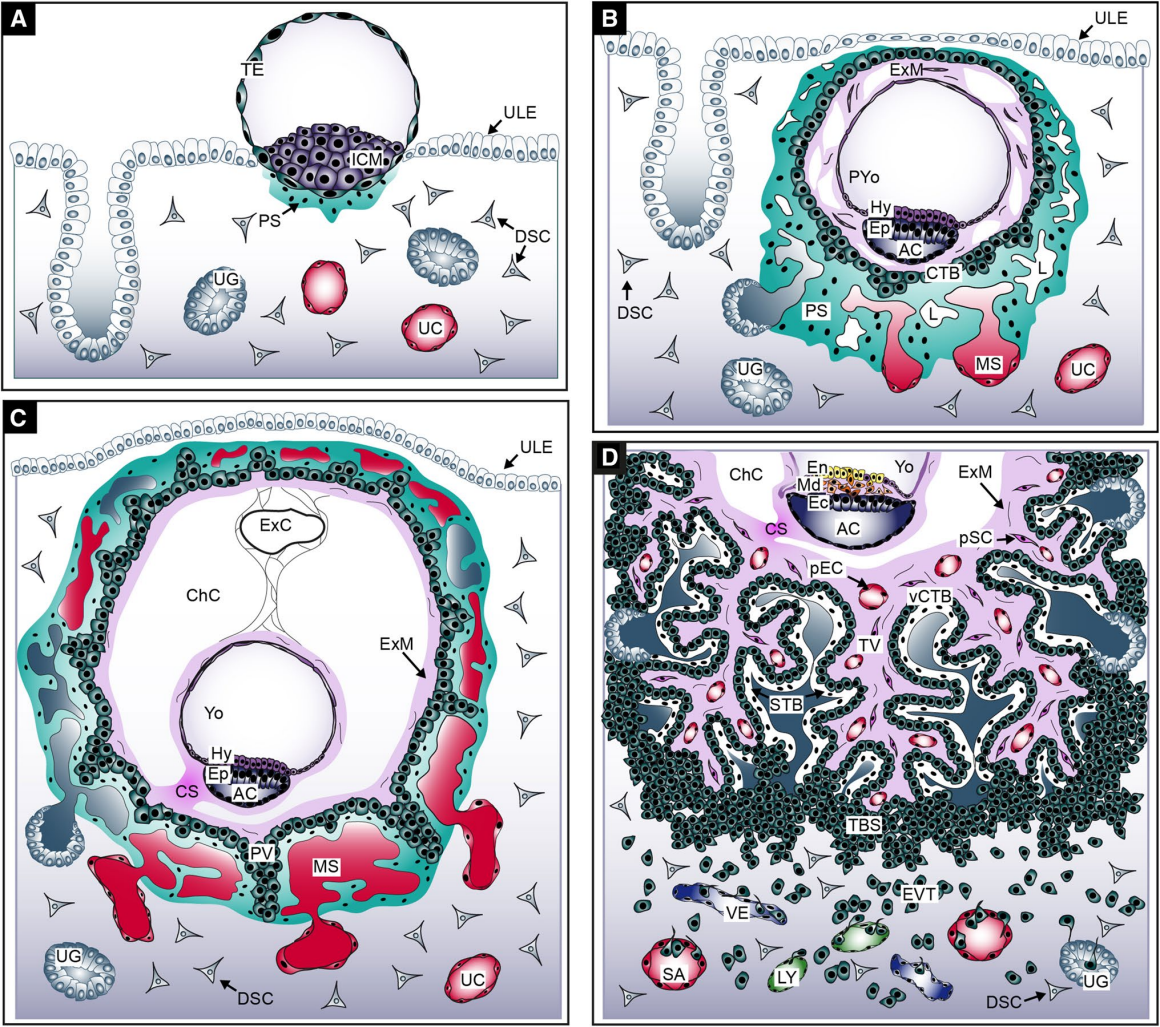
Development of the human placenta during the first 3 weeks of gestation. **a** Human blastocyst implanting into the pregnant uterus. **b** Development of the first placental structures and the embryonic disc. **c** Formation of primary villi and yolk sac. **d** Development of tertiary villi and the embryonic germ layers. *AC* amniotic cavity, *CS* connecting stalk, *ChC* chorionic cavity, *CTB* cytotrophoblast, *DSC* decidual stromal cell, *Ec* ectoderm, *En* endoderm, *Ep* epiblast, *EVT* extravillous trophoblast, *ExC* exocoelomic cyst, *ExM* extraembryonic mesoderm, *Hy* hypoblast, *ICM* inner cell mass, *L* lacunae system, *LY* lymphatic vessel, *Md* mesoderm, *MS* maternal blood sinusoid, *pEC* placental endothelial cell, *PS* primitive syncytium, *pSC* placental stromal cell, *PV* primary villi, *PYo* primitive yolk sac, *SA* spiral artery, *TBS* trophoblastic shell, *TV* tertiary villi, *UC* uterine capillary, *UG* uterine gland, *ULE* uterine luminal epithelium, *VE* venous vessel, *vCTB* villous CTB, *Yo* yolk sac

Our knowledge about the first weeks of human placental development (Fig. 1) is largely based on the interpretation of anatomical structures of early implantation sites present in hysterectomy specimens of the Boyd (Centre of Trophoblast Research, Cambridge) and Carnegie (Human Developmental Anatomy Center, Washington DC) collections [1, 43]. In addition, pre-implantation and implantation studies in mice [44, 45], as well as histological analyses of species with comparative placental development, such as the great apes [46, 47], contributed to our understanding of early placentation events in humans. The precursor of all trophoblast cells is the trophectoderm (TE) constituting the outer layer of the human blastocyst. The TE develops approximately 4–5 days after fertilization. Its formation represents the first lineage decision during development, segregating the TE from the inner cell mass (ICM), the latter giving rise to the embryo proper (Fig. 1a). Interaction of the so-called polar TE, adjacent to the ICM, with the uterine luminal epithelium results in implantation around day 6–7 post-conception at which time the first steps of placental development commence.

After implantation, stem cells of the TE (TESC) generate the first trophoblast lineages, early mononuclear cytotrophoblasts (CTBs) and the multinuclear primitive syncytium (PS) at day 8 post-conception [32, 48, 49]. The PS represents the first invasive placental cell type which further expands into the maternal decidua (Fig. 1b). At this time the ICM simultaneously develops into a bilaminar epithelial structure consisting of epiblast (Ep) and hypoblast (Hy; also termed primitive endoderm), giving rise to the embryo and the primitive yolk sac (pYO), respectively. Lineage tracing studies in primates show that the Hy also gives rise to the extraembryonic mesoderm (ExM), which in turn forms the mesenchymal compartment of chorionic villi and the umbilical cord [50]. However, the Ep may also contribute to the ExM, as ExM cell express markers traditionally associated with this lineage [51]. Around day 15 post-conception the Ep forms the three embryonic germ layers and the amnion. Approximately at day 9 vacuoles appear in the PS, which upon fusion form a network of lacunar spaces eventually breaching the maternal uterine capillaries (UC) around day 12–13 thereby forming discontinuous maternal blood sinusoids (MS) [1]. Around day 10 post-conception the development and morphogenesis of placental villi commences. At the time of PS expansion, rows of proliferative CTBs break through the expanding syncytial mass thereby forming primary villi (PV) (Fig. 1c). The PV extend into the underlying maternal decidua and, like the early multinuclear structures, erode uterine blood vessels and glands (UG). During the following days PV are transformed into secondary villi, achieved by migration of ExM cells into the primary structures. Concurrently, the epithelial surface branches and expands tremendously by continuous proliferation and cell fusion of developing villous cytotrophoblasts (vCTB). The latter process generates the outer multinuclear syncytiotrophoblast (STB) layer, providing the interface between mother and fetus for nutrient transport and gas exchange in floating villi. The STB is thought to arise from asymmetrical cell division, differentiation and fusion of villous cytotrophoblasts (vCTBs) with the pre-existing syncytium and secrete critical pregnancy hormones into the maternal circulation, such as human chorionic gonadotrophin (hCG) and placental lactogen [52, 53].

Around day 17 post-conception secondary villi develop into tertiary villi (TV) that contain placental vessels, at a time when the fetal allantois extends and fuses with the chorionic plate at later stage (Fig. 1d). These vessels begin as haemangiogenic foci which differentiate from the ExM. These haemangiogenic foci develop into primitive endothelial tubes. The recruitment of pericytes stabilizes these tubes allowing further expansion of the placental vascular network via increases in capillary length and diameter finally connecting placental vessels with the vasculature of the fetus after the fourth week of pregnancy [3]. Interestingly, the placenta leads the way in vascular development in the embryo, with the first blood vessels evident when the embryo proper still exists as three germ layers [54]. Consequently, all of the cell lineages involved in early placental haemangiogenesis and vasculogenesis are thought to arise in the placenta de novo via differentiation directly from the ExM, as the umbilical circulation does not connect the fetal and placental systems until 32 days post-conception. The placental vasculature continues to undergo extensive expansion the late-first and second trimester as a result of branching angiogenesis. Towards the end of pregnancy the placental capillaries elongate and form loops that are pushed up against the STB layer of terminal villi, decreasing the exchange distance between the maternal and fetal circulations and thereby maximizing oxygen and nutrient transport to the fetus [55].

**Fig. 2:**
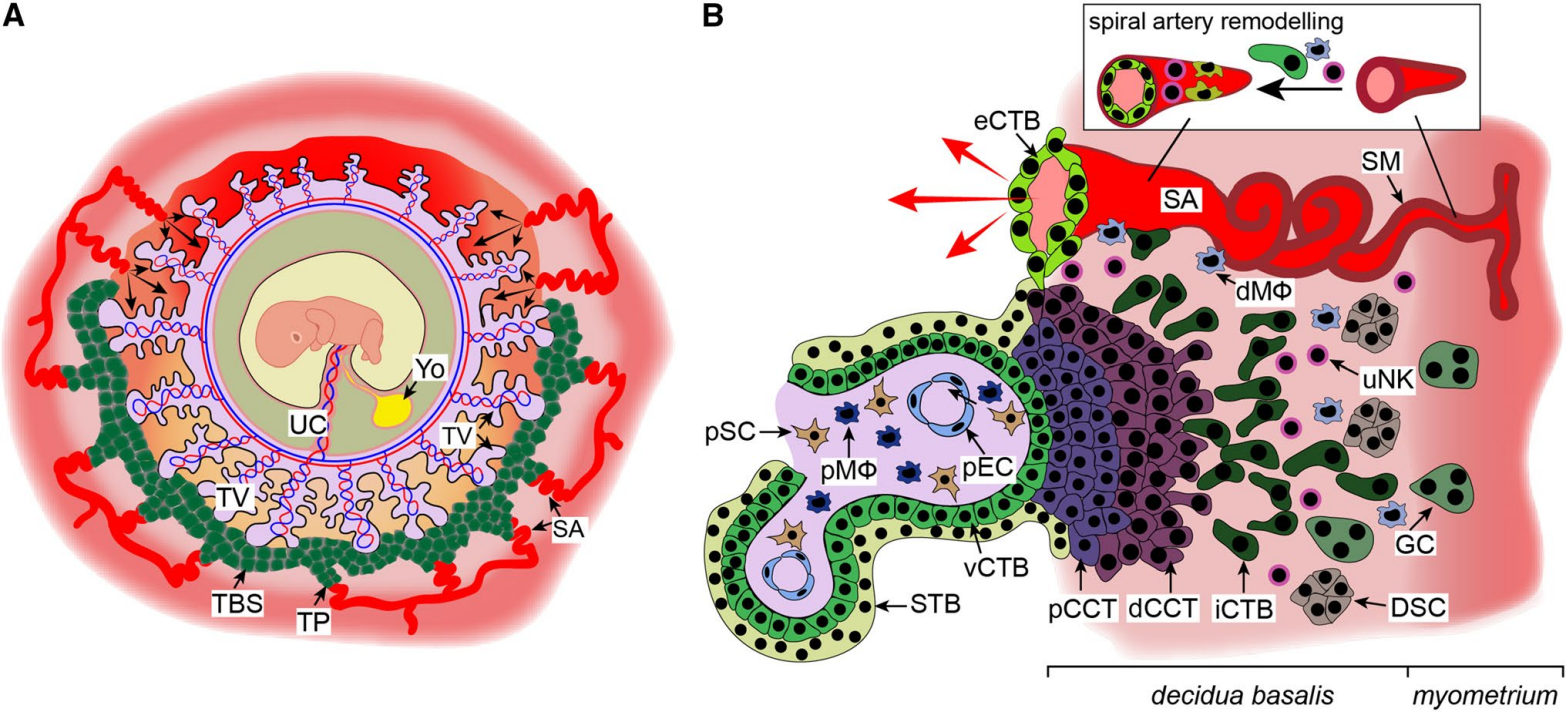
Development of the trophoblastic shell and formation of placental anchoring villi. **a** Structure of the human trophoblastic shell and its surrounding arterial vessels. **b** Depiction of a placental anchoring villus, spiral artery (SA) remodelling and interaction of extravillous trophoblasts (EVTs) with different decidual cell types. *dCCT* distal cell column trophoblast, *dMΦ* decidual macrophage, *DSC* decidual stromal cell, *eCTB* endovascular cytotrophoblast, *GC* giant cell, *iCTB* interstitial cytotrophoblast, *pCCT* proximal cell column trophoblast, *pEC* placental endothelial cell, *pMΦ* placental macrophage, *pSC* placental stromal/mesenchymal cell, *SM* smooth muscle layer, *STB* syncytiotrophoblast, *TBS* trophoblastic shell, *TP* trophoblast plug, *TV* tertiary villi, *UC* umbilical cord, *uNK* uterine NK cell, *vCTB* villous cytotrophoblast, *YO* yolk sac

Besides developing chorionic villi, proliferating CTBs at distal sites also expand laterally around day 15 post-conception to form the trophoblastic shell, which represents the outermost site of the placenta encircling the embryo (Fig. 2a). The shell lacks maternal cell types and is thought to be critical for anchorage of the placenta to the decidua and protection of the embryo from oxidative stress [56]. During the early phases of placentation the trophoblastic shell gives rise to the second differentiated trophoblast cell type, the invasive extravillous trophoblasts (EVTs). However, once mature placental villi have formed, EVTs originate from the differentiation of CTBs in the tips of anchoring villi (Fig. 2b). In these villi, rows of proliferative proximal cell column trophoblasts (pCCTs) develop, representing the progenitor cell pool of differentiated EVTs. Upon formation of the distal cell column, cells cease mitosis, but do not exit the cell cycle to reach a quiescent state. Instead, dCCTs enter endoreduplicative cycles and undergo polyploidization and senescence upon differentiation into EVTs [57]. By 15–16 days post-conception two distinct EVT populations can already be identified; interstitial cytotrophoblasts (iCTBs) invade the decidual stroma, whereas endovascular cytotrophoblasts (eCTB) colonize the maternal spiral arteries [58, 59]. Invasion of uterine stroma by iCTBs provokes numerous effects during early pregnancy. These cells interact with decidual stromal cells, macrophages and uterine natural killer (uNK) cells (Fig. 2b) in order to regulate immunological acceptance of the placental/fetal allograft and control EVT function [60, 61]. Besides migration into the spiral arteries, iCTBs also invade decidual glands, lymphatics and veins (Fig. 1d) [62–64]. Whereas breaching of glandular structures could be required for early histiotrophic nutrition of the embryo, invasion of lymphatics and veins might be necessary for fluid drainage, adaptation of immune cell trafficking and hormonal adaption to pregnancy. Indeed, EVT-specific proteins, such as diamine oxidase (DAO), are detectable in the serum of pregnant mothers prior to the onset of the maternal–placental circulation [65]. As iCTBs reach the underlying myometrium, they undergo a final differentiation step into multinucleated trophoblast giant cells losing their invasive capacity.

Migration of EVTs into the maternal spiral arteries represents another key step of human placentation (Fig. 2b). In early pregnancy these vessels are extensively remodelled within the decidua and as far as the first third of the myometrium. To achieve this, iCTBs are recruited to the spiral arteries by uNK cells and macrophages, which surround these vessels from early pregnancy and initiate the remodelling process [66, 67]. iCTBs then breach the spiral arteries, and differentiate into eCTBs that migrate along their lumen and adopt a vascular adhesion phenotype that allows them to interdigitate into the endothelial layer, whereby they induce endothelial cell apoptosis and completely replace the maternal endothelial cells within these vessels [68]. Concurrently, iCTBs induce apoptosis or dedifferentiation of the smooth muscle layer and basal lamina of the spiral arteries, thereby contributing to vessel remodelling [67, 69, 70]. This results in a dramatic change in the spiral arteries during which narrow vessels with relatively high resistance are transformed into highly dilated, low-resistance conduits (Fig. 2b). These remodelled vessels change the nature of blood flow entering the intervillous space later in pregnancy to ensure that the increase in volumetric blood flow to the uterus during pregnancy is delivered at an appropriately low speed to ensure maximal perfusion and prevent damage to the villi.

As well as remodelling the spiral arteries, eCTBs also form trophoblast plugs during the first weeks of pregnancy that occlude the spiral arteries in the decidua basalis underlying the implantation site (Fig. 2a). These plugs completely prevent blood flow until 6–7 weeks of gestation, after which narrow channels in the plugs begin to form, which may allow a limited flow into the intervillous space that can be detected by Doppler ultrasound [71]. Trophoblast plugs disintegrate completely near the end of the first trimester, and this is associated with significant onset of flow of oxygenated maternal blood into the intervillous space around 12–13 weeks of gestation [71–73]. This significant increase in flow is also thought to coincide with the completion of trophoblast-independent remodelling of the upstream radial arteries, which may act as the rate-limiting vessels regulating the volumetric flow of maternal blood into the intervillous space [71, 74–76]. As a result of trophoblast plugging, the placenta exists in a low oxygen environment for the majority of the first trimester, and this is thought to be key to promote placental development, vasculogenesis and angiogenesis. Indeed, a premature rise of oxygen levels could provoke production of reactive oxygen species and, as a consequence, oxidative damage of the fetal–placental unit, as incomplete plugging of the maternal arteries and disorganized early onset blood flow has been noticed in miscarried pregnancies [9, 11]. At the margin of placenta, where high oxygen flow is first initiated, the trophoblast layer may degenerate (Fig. 2a), providing a potential mechanism for the regression of villi and formation of the mature discoidal shape of the placenta [30]. The establishment of a vascular connection between the mother and fetus by the end of the first trimester marks the transition from histiotrophic to haemotrophic nutrition. In humans, the placenta is accordingly defined as haemochorial since placental villi are in direct contact with maternal blood filling the intervillous space.

## In vitro formation, identity and molecular control of differentiated placental trophoblast subtypes

Throughout the past few decades, research with primary trophoblasts has been dependent on the availability of placental tissues from different stages of pregnancy. Samples are most easily obtained after delivery of normal term pregnancies and of pregnancy disorders at late stages. However, at the end of gestation it is impossible to determine if placental pathology is the cause or the consequence of a given pregnancy complication. Moreover, distinct trophoblast functions, for example invasiveness and motility of EVTs, are significantly reduced at term [77]. Hence, among the different developmental processes of trophoblasts, only STB formation in vitro can be effectively studied using human term placenta. CTBs from term placentae isolated by trypsin digestion and Percoll gradient centrifugation spontaneously fuse into multinuclear structures when seeded on plastic or extracellular matrix (ECM)-coated dishes and upregulate markers of STB-identity such as hCG [78, 79]. Using this model numerous soluble factors, such as epidermal growth factor (EGF), as well as key regulatory genes promoting trophoblast syncytialization have been characterized [80–83]. Several trophoblast-secreted proteins increase cell fusion and transcription of hormone genes through elevation of cAMP levels, the latter activating crucial regulators in STB, such as cAMP-responsive element binding protein (CREB) and glial cells missing (GCM1) [82, 84, 85]. Notably, the villous trophoblast epithelium also expresses the fusogenic proteins syncytin-1 and -2, encoded by the human endogenous retroviruses (HERV) HERV-W and HERV-FRD, interacting with their respective receptors, the sodium-dependent neutral amino acid transporters (ASCT1 and ASCT2) and major facilitator superfamily domain containing 2a (MFSD2a), respectively [86]. Syncytin expression is controlled by a placenta-specific enhancer-binding GATA-binding proteins and GCM1 [87, 88]. The latter is critical for branching morphogenesis and syncytialization in mouse placenta, and was also shown to increase cell fusion of human CTBs [89, 90]. Additionally, other transcriptional regulators, such as activating enhancer-binding protein 2α (AP-2α), distal-less homeobox 3 (DLX3) and peroxisome proliferator activated receptor gamma (PPAR-γ), regulating hormone expression and syncytialization of term trophoblasts, have been identified [38, 91]. However, to what extent molecular mechanisms of cell fusion may differ between first and third trimester remains largely unknown. DNA microarray and RNA-seq data of early and late STBs, generated by in vitro cell fusion or analysed by single-cell sequencing of whole placental tissues, have been published [34, 92–95]. Yet, functional fusion studies with first trimester primary vCTBs are rarely performed due to the restrictions on using this material in many laboratories, and the limited amount of placental tissue obtained. Likewise, regulation of early PS formation remains enigmatic. Possibly STB, generated by treatment of human embryonic stem cells (hESC) with bone morphogenetic protein 4 (BMP4), could be representative of the early PS, since gene expression in that model is different to that of term STB [96].

Signalling pathways and key mechanisms controlling EVT migration, invasion and function have been widely investigated [97, 98]. Due to the limited availability of first trimester placental tissues different trophoblast cell lines have been utilized as cellular model systems [99]. However, most of these cell lines differ considerably from primary EVTs with respect to gene expression patterns and human leukocyte antigen (HLA) profiles [100, 101], questioning their origin and suitability as EVT models. In contrast invasive trophoblasts, isolated from first trimester placenta, express the correct HLA genes and cell-specific markers of the in situ EVT. In vitro, EVTs can be generated from purified CTBs or villous explant cultures, the latter recapitulating cell column proliferation and differentiation [102, 103]. After attachment to matrix, purified CTB cultures spontaneously induce markers of the migratory trophoblast, for example HLA-G, proteoglycan 2 (PRG2), erythroblastic oncogene B2 (ErbB2) and the EVT-specific proteins integrin α1 (ITGA1) and α5 (ITGA5) [64, 104–106], and upregulate matrix-metalloproteinases and other proteolytic enzymes for invasion into the decidua [107, 108]. Several transcription factors, including GCM1, AP-2α, signal transducer and activator of transcription 3 (STAT3), and FOS like 1 (FOSL1), were shown to control trophoblast invasion and EVT-specific gene expression in different trophoblast cell models [90, 109–111]. Furthermore, within the early placenta hypoxia-inducible factor 1 (HIF-1) is only expressed by EVTs, and low oxygen levels promote in vitro EVT formation [112] and elevation of the EVT progenitor marker Notch1 [113]. Another critical pathway regulating EVT function is canonical Wnt signalling [114]. Activity of nuclear Wnt-dependent T cell factor 4 (TCF4)/β-catenin complexes is induced during EVT formation and silencing of TCF4 impaired motility and EVT-marker expression [115, 116].

Although many trophoblast-specific transcription factors have been tested in the context of CTB proliferation and motility [38, 97], their specific roles in commitment and differentiation of the invasive trophoblast lineage remain elusive. This is explained by the fact that, until recently, self-renewing cell culture models were lacking and CTB preparations (containing EVT progenitors) rapidly differentiate when seeded in 2-dimensional (2D) layers on ECMs. In 2018, however, culture conditions for induction of the EVT lineage have been established in long-term expanding trophoblast models [33, 34].

EVT formation could be largely driven by an autocrine differentiation program operating independently of the surrounding environment. Besides spontaneous 2D differentiation in vitro, ectopic trophoblast of tubal pregnancies and anchoring villi, implanted into the kidney capsule of SCID mice, induce EVT markers and switch their integrin expression like EVTs invading the decidua basalis [117, 118]. Notably, CTBs derived from preeclamptic placental tissues show defects in EVT differentiation, suggesting that failures in this process could contribute to abnormal placentation in this disease [119]. On the other hand, EVTs isolated from preeclamptic tissues were shown to revert their gene expression pattern back to normal [120], suggesting that the decidua could play a role in shaping EVT function. These distinct results could possibly be explained by the different molecular subtypes of preeclampsia which may or may not have abnormal placentation as an underlying cause [20, 22, 121]. Despite the limited life-span of 2D primary cultures, in vitro EVT formation and differentiation also occurs in discrete steps as it has been described in vivo. Whereas many EVT-markers, such as HLA-G, ITGA5 or TCF4 are induced in the distal, non-mitotic part of the cell column, ITGA1 and DAO, for example, are expressed in deeper regions of the decidua where EVTs have detached from anchoring villi [65, 104]. Notably DAO, induced in pure CTB cultures in an autocrine fashion, appears at later stages of in vitro differentiation compared to HLA-G [65]. These data suggest that the different steps of EVT differentiation can be recapitulated in 2D CTB cultures.

DAO expression in situ is mostly detectable in a subset of EVTs approaching veins and arteries [65] suggesting that the DAO-secreting cells could represent another specific EVT subtype. Along these lines, single-cell RNA-seq of first trimester placental/decidual tissues revealed between one and three different EVTs signatures, depending on the respective analyses [93–95, 122, 123]. Likewise, varying results with respect to the numbers of vCTB (1–3), placental macrophage (1–2) and stromal cells populations (2–3) were obtained in the RNA-seq analyses [93–95, 122, 123]. The diverging data could be explained by differences in gestational age of placental samples, variations in the methodology (direct RNA-seq of single cells after enzymatic digestion of tissues vs. RNA-seq of HLA-G purified cells), different cell cycle phases of the populations, as well as vast differences in the numbers of sequenced placental single cells (ranging from dozens up to several thousands). Whereas these analyses could be helpful to identify novel (surface) markers of specific placental subtypes, their origin and developmental regulation remain elusive warranting further functional investigations. For example, whether putative EVT subtypes are specified by an intrinsic developmental program of the anchoring villus, or merely represent phenotypic differences induced by the different components within the decidua remains unknown.

## Origin, localization and identity of trophoblast stem and progenitor cells

CTB preparations from early pregnancy placentae undergo both cell fusion and EVT formation, and as a result CTBs were thought to be a homogenous bi-potential ‘stem cell-like’ population [124]. However, others found that EVT and STB develop from different subpopulations of vCTB, and that traditional CTB isolates contain both vCTBs and CCTs. In vivo, STBs are formed from the vCTBs that reside in a monolayer around the majority of the villus, and pure vCTBs can only form STBs in culture [113]. In contrast, precursors of the EVT lineage (CCTs) reside in proliferative multilayered clusters within villus tips and proximal cell columns [102, 125]. The notion that CTBs were not a homogenous bi-potential population was first raised by findings that sequential trypsinization led to the isolation of trophoblasts with different properties [126], and this was supported by subsequent findings that multilayered clusters of cells in the tips of first trimester villus explants were exclusively able to produce EVT outgrowth, but not regenerate the STB [127]. This EVT progenitor population was subsequently isolated and, unlike standard CTB preparations, proliferated slowly in culture, with around 20% of cells differentiating into HLA-G positive EVTs, and no evidence of STB differentiation [125]. The subsequent development of alternative methods to isolate highly purified CCTs, has allowed much higher rates of EVT differentiation (> 90%) to be achieved, and has enabled a greater understanding of the signalling pathways that distinguish EVT and STB progenitor populations [113].

The presence of distinct progenitors for EVT and STB means that vCTBs should not be considered a ‘stem cell’ population, and rather that a less differentiated ‘true’ trophoblast stem cell (TSC) population, that acts as the precursor to both progenitor populations, must reside within placental villi. Such a TSC population would overcome the limitations of many traditional primary trophoblast models such as heterogeneity between different cell isolations, and the limited lifespan of isolates in culture, and thus has been an intense focus of the field over the past decade. TSC populations have previously been isolated from pre-implantation blastocysts in a number of animal models (porcine, bovine, rhesus monkey, and murine), of which murine TSCs are the best characterized [128–130]. Murine TSCs can be maintained in an undifferentiated state in the presence of fibroblast growth factor 4 (FGF4) and heparin, whilst removal of FGF4 from these cultures induces their differentiation into cells of the extraembryonic ectoderm, ectoplacental cone, and trophoblast giant cells [131].

Despite our knowledge of TSCs from animal models, the isolation of human TSCs proved considerably more challenging, and as a result was not achieved until recently. This difficulty in deriving human TSC may have in part arisen from the distinct anatomical differences between the human placenta and that of many animal models, as neither markers critical to murine TSC self-renewal, or culture conditions used to propagate these cells, proved to be transferable to the human TSC derivation [132]. Indeed, key differences in the expression of lineage-associated factors have been observed between species [133, 134] as discussed below.

The recent establishment of a human TSC model will allow us to begin to truly unpick these species-specific differences to understand the unique nature of human trophoblast lineage differentiation in the mammalian context, and will play an important role in translating data between species. The first human TSC population was isolated by enzymatically digesting first trimester placental villi, purifying α6 integrin-positive vCTBs, and then culturing these cells in a novel medium formulation that maintained these vCTB in culture far longer than had previously been possible [33]. After several passages, these cultures were taken over by the proliferative subset of TSCs, allowing the establishment of TSC lines that could be maintained in an undifferentiated state for up to 5 months [33]. Similar populations were also established from the TE outgrowths of human blastocysts [33]. However, the significant breakthrough in this paper arose from the authors’ ability to identify conditions in which the human TSC within vCTB preparations could be expanded long-term (discussed below). Transcriptomic analysis revealed that human TSCs express critical markers of trophoblast identity and self-renewal [33], such as cytokeratin-7, GATA3, TEA-domain transcription factor 4 (TEAD4) and tumour protein p63 (TP63) [135, 136]. However, markers associated with murine TSCs were either absent or weakly expressed by human TSCs (or any other primary human trophoblast isolates examined) [33], suggesting that different transcriptional networks could be important for human trophoblast development.

To date, TSCs have not been able to be isolated from third trimester placentae [33, 34]. However, the ability to do this would provide a significant advance by enabling researchers to link TSC function to pregnancy pathologies in order to understand how these pathologies may have developed. Furthermore, this model of TSC derivation requires prolonged culture, which may lead to adaptation to in vitro conditions that may mask important differences between normal and pathological tissues. Therefore, whilst this has provided a quantum leap forward in our ability to investigate human TSCs, work remains to be done to identify unique cell surface markers of this TSC population, or ways to isolate these cells directly from fresh placental tissue of normal and pathological pregnancies.

Isolation of human TSCs was attempted for many years. Indeed, a number of candidate TSC populations were in existence prior to the breakthrough publication by Okae et al., and it will be of interest in the future to determine how these populations respond to the TSC-specific culture conditions defined by these researchers. The first significant potential candidate human TSC population in the literature was isolated after trypsin digestion of first trimester chorion from which the villi had been removed [137]. When cultured on gelatin, this ‘trophoblast progenitor’ (TBPC) population expressed POU class 5 homeobox 1/OCT4, cytokeratin-7 and GATA4, but lacked expression of more differentiated trophoblast markers including GCM1 and hCG [137]. Culture on Matrigel provoked differentiation, resulting in down-regulation of OCT4, and induction of STB and EVT markers [137]. However, more recent work demonstrates that these cells predominantly differentiate into EVTs, suggesting that they are more akin to an EVT progenitor population than a true human TSC [138].

Several groups have attempted to isolate human TSC populations by exploiting a characteristic of many adult stem cell populations; the ability to rapidly efflux Hoechst 33342, resulting in a characteristic ‘streak’ of low intensity staining (termed a side-population) when analysed by flow cytometry [139, 140]. As the trophectoderm expresses 90-fold more of the primary Hoechst efflux pump ABCG2 than ICM derived hESCs, the side-population technique is particularly promising for human TSC isolation [141]. Takao et al. identified a side-population within both primary first trimester CTBs (constituting 0.12% of cells) and the HTR8/SVneo cell line (constituting 0.53% of cells) that uniquely co-express interleukin 1 receptor type 2 (IL1R2) and interleukin 7 receptor (IL7R) [142]. More recently, the side-population technique was combined with a novel trophoblast isolation protocol to isolate a candidate human TSC population from first trimester villous tissue that is distinct from both TBPC and the side-population isolated by Takao et al. [143]. These side-population trophoblasts form a distinct population more closely related to vCTB than EVT at both the transcriptomic and methalomic level [143, 144]. Furthermore, these cells express markers that maintain the stem cell state including WNT5A, Kruppel-like factor 4 (KLF4) and OCT6, as well as markers involved with both murine and human trophoblast lineage differentiation, including E74-like ETS transcription factor 5 (ELF5), TEAD4, BMP4, and fibroblast growth factor 2 (FGF2) [143]. However, further work is required to confirm the stem cell status of side-population-derived candidate human TSC populations via their differentiation into the mature trophoblast lineages.

## Origin, localization and identity of stem and progenitor cells of the villous core

Whilst TSC populations are a key focus in the field, it is important to remember that trophoblasts in vivo do not exist or differentiate in isolation. Placental development is also crucially dependent on stem cell lineages within the villus core that play critical roles in influencing the morphogenesis of the branching architecture of the placenta, and in driving placental vascular development. Cells in the core of the placental villi arise from the ExM, which itself most likely originates from the Hy [145, 146]. In addition, progenitors from the yolk sac may migrate to the placenta, although these cells are more likely to colonize the placenta only after the umbilical circulation has opened up around 32 days post-conception [49, 147]. During very early placental development it is thus likely that the non-trophoblast lineages of the placenta arise de novo from sequential differentiation of the ExM. However, the exact lineage differentiation pathways, and the factors that regulate them, are only beginning to be understood.

The earliest progenitor of vascular lineages in the placenta that has been isolated to date is a population of CD43-, CD31- and CD144-positive cells akin to a mesenchymoangioblast population [148]. Mesenchymoangioblasts isolated from other tissues have the potential to differentiate into all major vascular lineages including endothelial cells, smooth muscle cells, pericytes and mesenchymal stem cells [149], and in a similar manner placental mesenchymoangioblasts have been shown to form colonies containing both mesenchymal and endothelial cells [148]. This provides a likely candidate from which cells of the placental blood vessels originate. However, the question remains as to where the haematopoietic lineages (Hofbauer cells and red blood cells) arise from, as they are evident in the placenta from 18 days post-conception, prior to the onset of the uterine circulation [54, 150]. It has been hypothesized that an even earlier precursor that could give rise to both mesenchymoangioblasts and haemangioblasts (from which blood lineages could derive) may exist in the early human placenta, although the relationship of this cell to the ExM that first invades the placental villi is unknown [49, 148]. Such a precursor could give rise to the early haemangiogenic foci, which may function in a similar way to haemangiogenic endothelium seen in other embryonic systems whereby red blood cells arise directly from the endothelial layer. Indeed, haematopoietic stem cells expressing RUNX1 (required for an endothelial-to-haematopoietic transition) have been identified in murine placentae, suggesting a similar population of cells may exist in human placentae [151, 152]. Despite their haematopoietic associations, Hofbauer cells are spatially isolated from haemangiogenic foci in early placentae [151]. Indeed, it seems that this population may arise from a precursor population identified within the placental stroma that exhibits a fibroblastic morphology, but expresses the macrophage/monocytic markers CD115 and CD14 [153].

Finally, mesenchymal stem cells (MSCs) themselves, which reside in a perivascular niche in the placenta throughout gestation, could directly contribute to the development of the placental vasculature via differentiation into endothelial cells and smooth muscle cells. Placental MSCs can be differentiated in vitro into cells that express a number of endothelial markers including von Willebrand factor (vWF), CD31, VEGFR2 and CD144, and their propensity to differentiate down this pathway is greater than MSC populations derived from bone-marrow, aligning with the concept that this is their biological function in vivo [154, 155]. However, whilst MSCs from other tissues are able to differentiate into smooth muscle cells and pericytes, to date the ability of placental MSC to differentiate into these cell types has not been demonstrated [49, 156]. The above suggests that while we are beginning to reveal distinct populations that may contribute to the development of cells within the villous core and vasculature, we still have little understanding of how the different progenitor populations within the core of the early placental villi relate to each other, nor of the different contributions these cells may make to the ongoing growth and development of the placenta across gestation. Future work to identify and propagate a progenitor cell at the apex of the placental core lineages may help to shed light on the relationships between these cell populations at both the functional and molecular level.

## Self-renewing model systems recapitulating placental development and differentiation

The absence of a human TSC for most of the past decade has led researchers to use alternative self-renewing model systems to mimic early placental development, the most popular of which has been human embryonic stem cells (hESCs). In vivo, cells of the ICM (from which most hESC lines are derived) form the embryo proper and not the placenta [157]. However, in vitro, BMP4 treatment can be used to induce hESC to differentiate into trophoblast-like cells [158, 159]. In such models, morphological differentiation into trophoblast-like cells is first observed at the periphery of cell colonies that display a flattened phenotype, but over time cell differentiation spreads inwards towards the colony centre [157, 160, 161]. Whilst the wide variation in exact differentiation protocols used (i.e. concentration, time, starting hESC line) can subtly vary the gene expression pattern [32], these models generally exhibit a down-regulation in the expression of pluripotency factors (NANOG and OCT4) and induction of caudal-related homeobox transcription factor 2 (CDX2), the master regulator of murine trophoblast development [162], provoking trophectoderm lineage differentiation [163–167]. However, this model is not without limitations, as it can produce mixed cultures that express trophoblast, mesodermal and vascular endothelial cell markers, suggesting that it does not specifically induce differentiation towards the trophoblast lineage [168–170]. Indeed, the generation of mixed mesodermal cultures from BMP4-treated hESCs has caused some authors to question whether BMP4 drives hESC differentiation into trophoblast-like or mesodermal-like cells [169–171]. As a result, hESC models of trophoblast differentiation have been more recently refined to prevent mixed phenotype cultures by inhibiting Activin/Nodal and FGF2 signalling pathways, resulting in cultures that are 80–100% trophectoderm/trophoblast-like with undetectable levels of the mesodermal marker Brachyury [167, 170]. TSC-like trophoblasts, co-expressing CDX2 and p63, have also been isolated from this model by using low doses of BMP4, which could be differentiated into STBs and EVTs in vitro [172].

Further, putative TSCs have also been established from early stages of embryonic development. The manipulation of mixed potency morula blastomeres has allowed the derivation of a human TSC line (USFB6) from a single blastomere of an eight-cell human embryo [173]. USFB6 cells maintain the expression of CTB markers when cultured in FGF2 with Activin/Nodal pathway inhibition and form EVTs and STB with an absence of mesodermal markers. However, USFB6 cells do have a more mesenchymal morphology than the human TSC population isolated by Okae et al., although it is unclear whether this is a consequence of the differing culture conditions the cells are maintained in [33, 173].

Human TSCs, self-renewing on collagen IV in 2D, have been established from blastocysts and first trimester CTB preparations [33]. In contrast to mouse TSCs, requiring FGF4 and transforming growth factor-β (TGF-β) signalling for maintaining stemness [131], treatment with EGF, and inhibition of histone deacetylase (valproic acid), Rho-associated protein kinase (Y27632), TGF-β signalling (A83-01) and glycogen synthase kinase-3 (CHIR99021) were sufficient for continuous proliferation of human TSCs [33]. CHIR99021 inhibits β-catenin degradation thereby activating canonical Wnt signalling, which besides its role in EVT differentiation [115, 116], seems to be critical for trophoblast self-renewal. Similar culture conditions were applied to develop long-term expanding 3-dimensional (3D) trophoblast organoids (TB-ORGs) from first trimester placental tissues. In the first paper published on the derivation of TB-ORGs Haider et al. used EGF, the TGF-β signalling inhibitor A83-01, the BMP signalling inhibitor Noggin, and the activators of Wnt signalling, R-spondin, CHIR99021 and prostaglandin E2, substances which have been utilized to successfully establish epithelial organoids from other human tissues [34, 174]. In a recent paper, confirming establishment of TB-ORGs, EGF, FGF2, A83-01, CHIR99021 and R-spondin were used [175]. In summary, these studies suggest that activation of Wnt and EGF signalling and inhibition of the TGF-β pathway could be sufficient for the derivation and long-term expansion of human TSCs and placental organoids.

TB-ORGs, growing in growth factor-reduced Matrigel, mimic the in vivo structure of human placenta, express markers of trophoblast identity and stemness and actively secrete pregnancy hormones [34, 175]. TB-ORGs exclusively contain trophoblast cells allowing researchers to study discrete steps of placental development in a simplified fashion. STBs and EVTs can be generated in both 2D TCSs and 3D TB-ORGs suggesting that the respective progenitors are maintained in vitro. Yet, conditions for STB formation need considerable improvement, to further identify key transcription factors and signalling molecules for STB commitment and differentiation. Syncytialization of 2D TCSs was shown to require elevation of cAMP levels by forskolin [33], a rather unspecific treatment which has been used for decades to fuse primary CTBs. In contrast, growing TB-ORGs undergo spontaneous fusion towards the centre [34, 175], rendering the current conditions unsuitable for controllable induction of the SBT lineage.

The EVT lineage could be induced in 2D TSCs by transiently adding neuregulin and soluble Matrigel and by elevating A83-01 in the absence of valproic acid and EGF [33], conditions that were later also applied to generate invasive trophoblasts in TB-ORGs [175]. However, utilization of these culture conditions remains unclear, since EVT progenitors lack neuregulin receptors consisting of heterodimers of ErbB2 and ErbB3 [105]. The latter are exclusively expressed on EVTs promoting their survival [105], and therefore cannot be required for commitment of the EVT lineage. Also, EVTs originating from 2D TSCs, display an unusual spindle-shaped morphology and lack critical markers such as insulin like growth factor binding protein 3 (IGFBP-3), pappalysin 1 (PAPP-A), DAO or PRG2 [33]. In contrast, others demonstrated that removal of the Wnt activator CHIR99021 was sufficient for induction of the EVT lineage in TB-ORGs [34]. Absence of the GSK-3 inhibitor produced Notch1-positive CCTs, the prime marker of EVT-progenitors [113], which further differentiated into EVTs expressing all commonly accepted markers for these cells. Differentiation also occurred in a spatial correct orientation suggesting that these culture conditions closely recapitulate in vivo cell column formation and EVT differentiation [34].

## Key regulatory transcription factors of human trophoblast development

Our present view regarding key regulators of human trophoblast development is based on their expression patterns in the placenta and a few functional studies in isolated primary trophoblast, villous explant cultures and trophoblast cell lines [38]. Transcription factors, playing a pivotal role in mouse trophoblast development and differentiation, have been unravelled [35, 176]. Therefore, speculation on the putative functions of their analogous genes in the human placenta can be elaborated. For these comparisons one must assume that proliferative spongiotrophoblasts and CCTs, and giant cells and EVTs, respectively, are equivalent cell types in the two species. Indeed, several key transcription factors, for example AP-2γ, encoded by the *TFAP2C* gene, or inhibitor of DNA binding 2 (ID-2), are expressed in proliferative CTBs and CCTs which, like their murine counterparts, could promote trophoblast development and proliferation [177, 178]. Others, such as achaete–scute family basic helix–loop–helix (bHLH) transcription factor 2 (ASCL2) and heart and neural crest derivatives expressed 1 (HAND1) are also expressed during human trophoblast development. However, their placental subtype-specific expression patterns differ between mouse and men. Whereas murine *Mash2/Ascl2* is a spongiotrophoblast-specific gene that controls maintenance and proliferation [179], *HASH2/ASCL2* is expressed in both cell columns and EVTs, suggesting that function of the human gene diverges [112, 180, 181]. *Hand1* is critical for murine giant cell formation, while it is absent from first trimester human placenta [182, 183]. However, the particular bHLH protein is expressed in the outer layer of human blastocysts and BMP4-treated hESC, indicating a possible role in early TE development [184, 185].

Furthermore, analyses of blastocysts and early gestational tissues revealed common expression patterns between murine and human TE and placenta [133, 186]. Some key factors, such as GATA3 controlling trophoblast self-renewal and differentiation [187], are present in both TE and placental trophoblast of mouse and men [188]. However, timing and expression of several critical regulatory genes differ between the two species [189]. The prime markers of murine TE and ICM specification, Cdx2 and Oct4, respectively, are restricted to the respective tissues in murine pre-implantation embryos and inhibit each other’s expression [162, 190]. However, these genes have been reported to be co-expressed in the TE of cultured human blastocysts at 5 days post-fertilization [188, 189, 191]. Compared to freshly flushed mouse blastocysts, co-localization of these key regulators in the human TE could either represent a functional difference between mice and men or be a consequence of the in vitro cultivation delaying downregulation of OCT4 in the TE compartment. Several critical genes of murine TE specification and self-renewal, such as eomesodermin (*Eomes*) and *Elf5* [192, 193], are absent from human TE [133], which could be key for a faster differentiation process in humans. However, ELF5 protein and/or mRNA were found to be expressed in vCTBs of the early human placenta and in self-renewing CTBs of TB-ORGs [34, 186, 194]. Additionally, its presence in pCCTs was suggested [194]. However, ELF5 protein expression could not be confirmed recently [186] and Notch1^+^ EVT progenitors, generated in TB-ORGs, considerably downregulated *ELF5* mRNA [34], questioning its role in cell column proliferation. Hence, human ELF5 could be mainly required for vCTB expansion after implantation and during morphogenesis of placental villi. In conclusion, a distinct set of regulatory genes might control human TE specification and maintenance, despite some overlap with mouse. Along those lines, AP-2γ, AP-2α, GATA2 and GATA3, expressed in human TE, were sufficient for the induction of CTB/TE-specific genes and repression of OCT4 in the BMP4-hESC model [195].

CDX2, the critical transcriptional regulator of murine TE specification, is also expressed by the human TE. However, discordant results with respect to its localization were published. Similar to mice, Kunath et al. observed nuclear expression in the TE of cultivated human blastocysts, whereas the factor was absent from the ICM [132]. In contrast, others found that CDX2 mostly localizes to the TE cytoplasm with variable levels between blastocysts [188, 189]. Therefore, further investigations are needed to clarify its localization and potential role in human TE development. In first trimester placenta CDX2 is present in the nuclei of vCTBs close to the chorionic plate, whereas no expression is seen in proliferative cell columns of the basal plate [113, 186]. Hence in the human placenta, similar to ELF5, CDX2 could have its main role after implantation by promoting TSC/CTB maintenance and proliferation. At subsequent developmental stages, and/or in regions distal from the chorionic plate, self-renewal of TSCs may not require CDX2, since only a few cells within TB-ORGs and 2D-TSCs weakly express the gene, despite the fact that these cultures give rise to STBs and EVTs. Another key transcription factor of mouse TE development, TEAD4, could fulfil this role. Controlled by co-activators of the Hippo pathway *Tead4* was shown to activate *Cdx2* expression and TE development in mice [196, 197]. However, its precise role in human CTBs remains to be elucidated. Human TEAD4 localizes to the nuclei of proliferative CTBs of 2D-TSCs and TB-ORGs, and of placental vCTBs in vivo, while its expression is downregulated in cell columns of anchoring villi [33, 34, 113, 186]. The latter might be achieved by CCT-specific induction of Notch1 in villi of the placental basal plate. Notch1 is present in proliferative pCCTs generating the transcriptional co-activator Notch1 intracellular domain (N1ICD) by γ-secretase-mediated cleavage from the cytosolic region of the receptor [113]. N1ICD promoted trophoblast survival, and repressed markers of vCTB stemness, i.e. TEAD4 and p63, the latter by promoting its degradation via upregulation of interferon regulatory factor 6 (IRF6), and induced the EVT progenitor-specific genes, MYC and VE-cadherin [113].

**Fig. 3:**
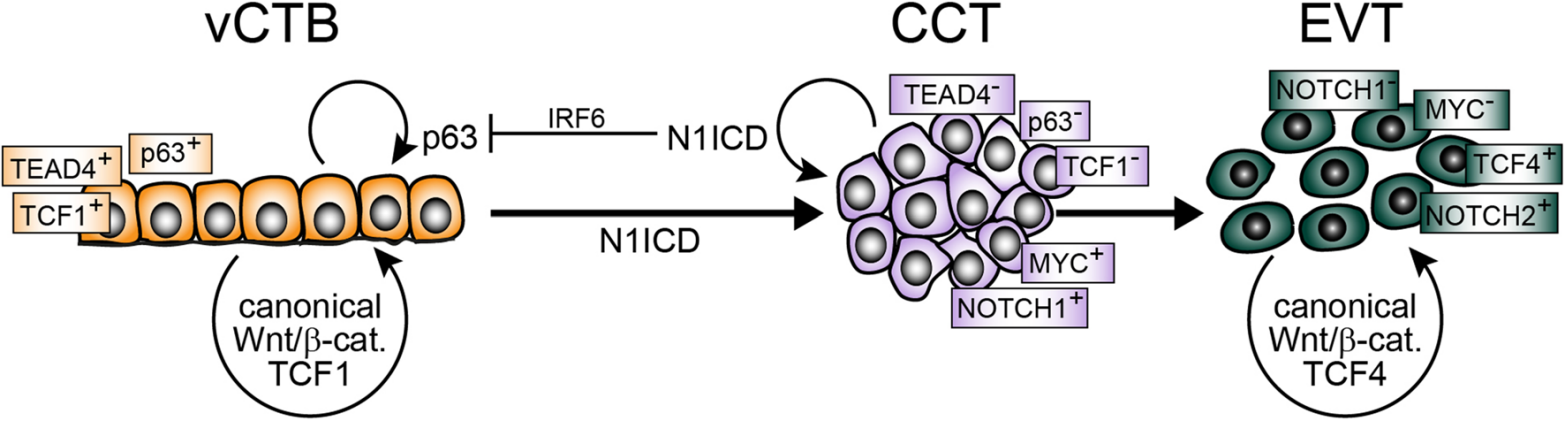
Model system integrating the role of Notch/Wnt signalling in trophoblast stemness and EVT differentiation. Notch1 intracellular domain (N1ICD) represses markers of villous cytotrophoblast (vCTB) self-renewal, i.e. TEAD4 and p63, and induces expression of the extravillous trophoblast (EVT)-progenitor-specific gene MYC. Formation of these precursors is also associated with the loss of TCF1 expression, whereas β-catenin–TCF4 complexes arise during EVT formation. *CCT* cell column trophoblast, *IRF6* interferon regulatory factor 6, *TCF* T-cell factor, *Wnt* wingless

So far, Notch1 represents the only functionally tested regulator for initiation of the EVT lineage (Fig. 3). It is expressed in a subset of cyclin A-positive pCCTs suggesting that the EVT progenitor cell pool could give rise to Notch1-negative transient-amplifying cells, which further differentiate into EVTs [113]. Noteworthy, pCCTs lack expression of TCF genes, suggesting that canonical Wnt signalling might not be critical for formation and/or maintenance of proliferative cell columns. Indeed, removal of Wnt activators promoted development of TCF-negative EVT-progenitors in TB-ORGs [34]. However, as these cells undergo differentiation, they induce Wnt-dependent transcription factors and nuclear β-catenin, likely as part of an epithelial to mesenchymal (EMT)-like program operating during EVT formation [115, 198, 199]. Therefore, canonical Wnt signalling could play a dual role in human placental development (Fig. 3). In analogy to its role in other stem cell niches [200], Wnt-activated TCF1, which is expressed in a subset of TB-ORGs and vCTBs [34], could be necessary for self-renewal of TSC. In contrast, signalling through TCF4–β-catenin complexes could promote EVT differentiation and function. A switch in Notch receptor expression across the anchoring villus might also be critical in this process, as Notch2 is induced in dCCTs and EVTs, controlling motility of the latter [201, 202].

So far, little is known about altered functions/mutations of trophoblast-specific transcription factors in pregnancy disorders. However, a specific genotype of the winged helix protein STOX1, controlling the balance between cell column proliferation and EVT invasion, was found to be associated with a familial form of severe preeclampsia [203, 204].

## Conclusions

**Fig. 4:**
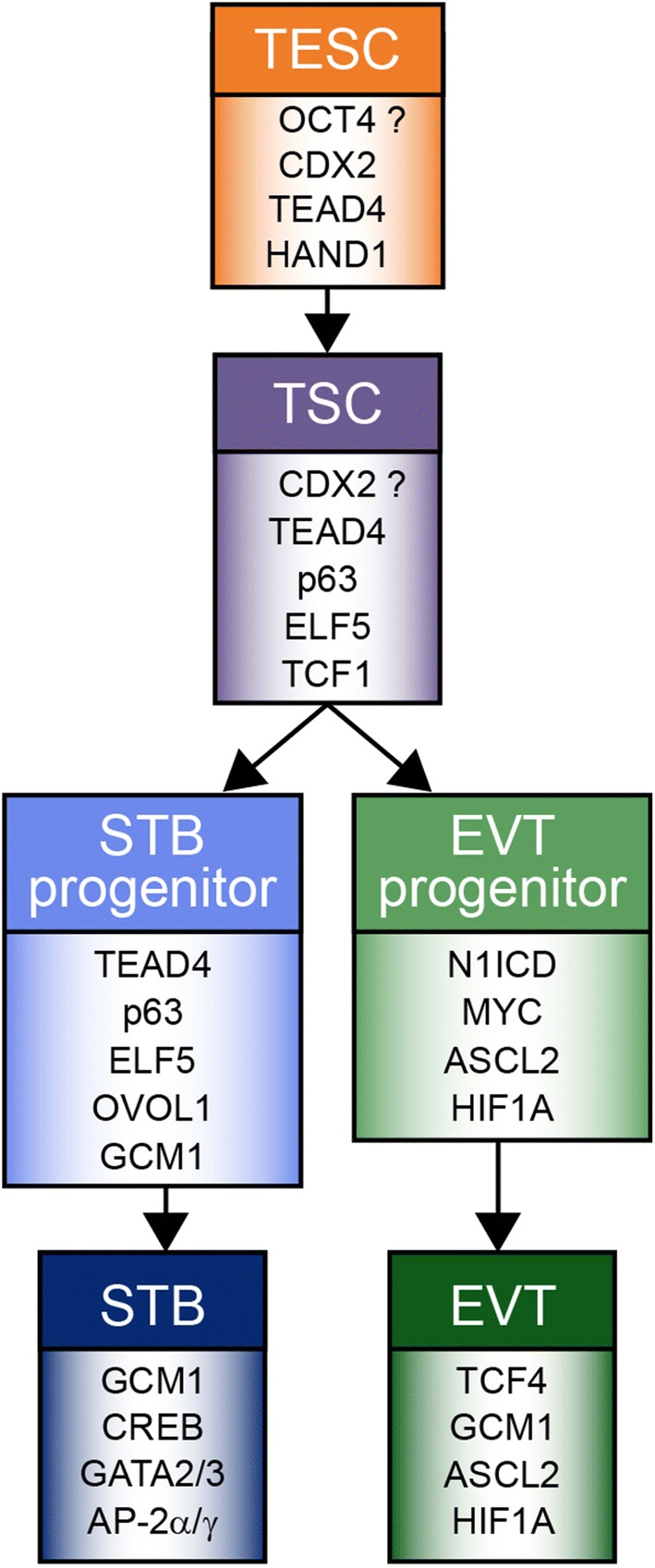
Trophoblast lineage development and key regulatory transcription factors expressed in the different trophoblast subtypes. Regulators, operating in both syncytiotrophoblast (STB) and extravillous trophoblast (EVT), are not depicted. Likewise, distal cell column trophoblasts, developing from EVT progenitors are not shown, since these cells express the same repertoire of key transcription factors as EVTs. Further differentiation steps of these cells in deeper regions of the decidua were omitted, since knowledge about the associated transcription factors is lacking. AP-2α, AP-2γ and GATA3 represent reliable markers of trophoblast identity, yet are present in most trophoblast subtypes. Herein, presentation of these factors only in STB aims indicating their predominant roles in placental hormone expression. A possible role of OCT4 in the pre-implantation trophectodermal stem cell (TESC) remains disputable. The absence of CDX2 from the majority of CTBs of self-renewing cultures, questions its role in post-implantation trophoblast stem cell (TSC) maintenance. TSCs are potentially equivalent to self-renewing villous cytotrophoblasts of the placental epithelium

Our current knowledge about human TSCs, their derivatives and specific key transcription factors predominantly relies on comparative expression patterns between mouse and human trophoblast. Based on these data and some functional studies, we herein speculate about putative markers discriminating the different human trophoblast subtypes (Fig. 4). Co-expression of CDX2, and TEAD4, among others such as GATA3 and AP-2γ, is characteristic for trophectodermal stem cells (TESC). However, critical key factors for the development of post-implantation TSCs/early self-renewing CTBs are unknown, since in situ expression data and appropriate model systems are lacking. Accordingly, putative differences between expanding CTBs of primary villi/trophoblastic shell and early proliferative vCTBs remain unknown. Possibly, induction of ELF5 and concomitant expression of CDX2 could be hallmarks of post-implantation TSCs. However, CDX2 is largely absent from vCTBs of distal villi of early placentae and its expression could not be maintained in self-renewing TB-ORGs at higher passage numbers [34, 186]. Hence, CDX2-positive cells of the first trimester human placenta could also represent residual TESC which might be distinct from self-renewing TSCs and require different culture conditions for long-term maintenance. Moreover, TCF1 could also mark TSCs due to its restricted expression in placental villi and its known role in other stem cell niches. Further, we postulate that precursors within the trophoblast epithelium, prone to fusion, could express a different set of pivotal regulators, as compared to self-renewing TSCs. Whereas ELF5, TEAD4 and p63 are present in all first trimester vCTBs, ovo-like transcriptional repressor 1 (OVOL1) and GCM1 are restricted to a subset of progenitors in which STB formation could be initiated. Accordingly, both GCM1 and OVOL1 were shown to promote STB formation, the latter by repressing stemness- and proliferation-associated genes [205]. N1ICD has been identified as a master regulator of EVT lineage induction inhibiting vCTB stemness genes. Hypoxia and contact to the decidual matrix could be critical triggers of EVT lineage commitment and differentiation, respectively. Numerous transcription factors, controlling migration and invasion, are induced in EVTs in vivo and during 2D differentiation of primary CTBs. However, their specific role in developing EVT progenitors awaits further studies in the recently established, self-renewing trophoblast models.

In summary, the previous absence of expandable systems hampered deciphering key steps of human trophoblast development. However, establishment of self-renewing cultures should allow delineating pivotal regulators of human placentation in the near future. Moreover, in vitro modelling of pregnancy complications, for which failures of trophoblast growth and differentiation could be underlying causes, should be advanced by establishing 2D-TSCs and TB-ORGs from abnormal placental tissues.
